# Supplementary figures and images for: NetSHy: network summarization via a hybrid approach leveraging topological properties
Source: Bioinformatics. 2022 Dec 22;39(1):btac818. doi: 10.1093/bioinformatics/btac818 (PMC9831052; doi:10.1093/bioinformatics/btac818)

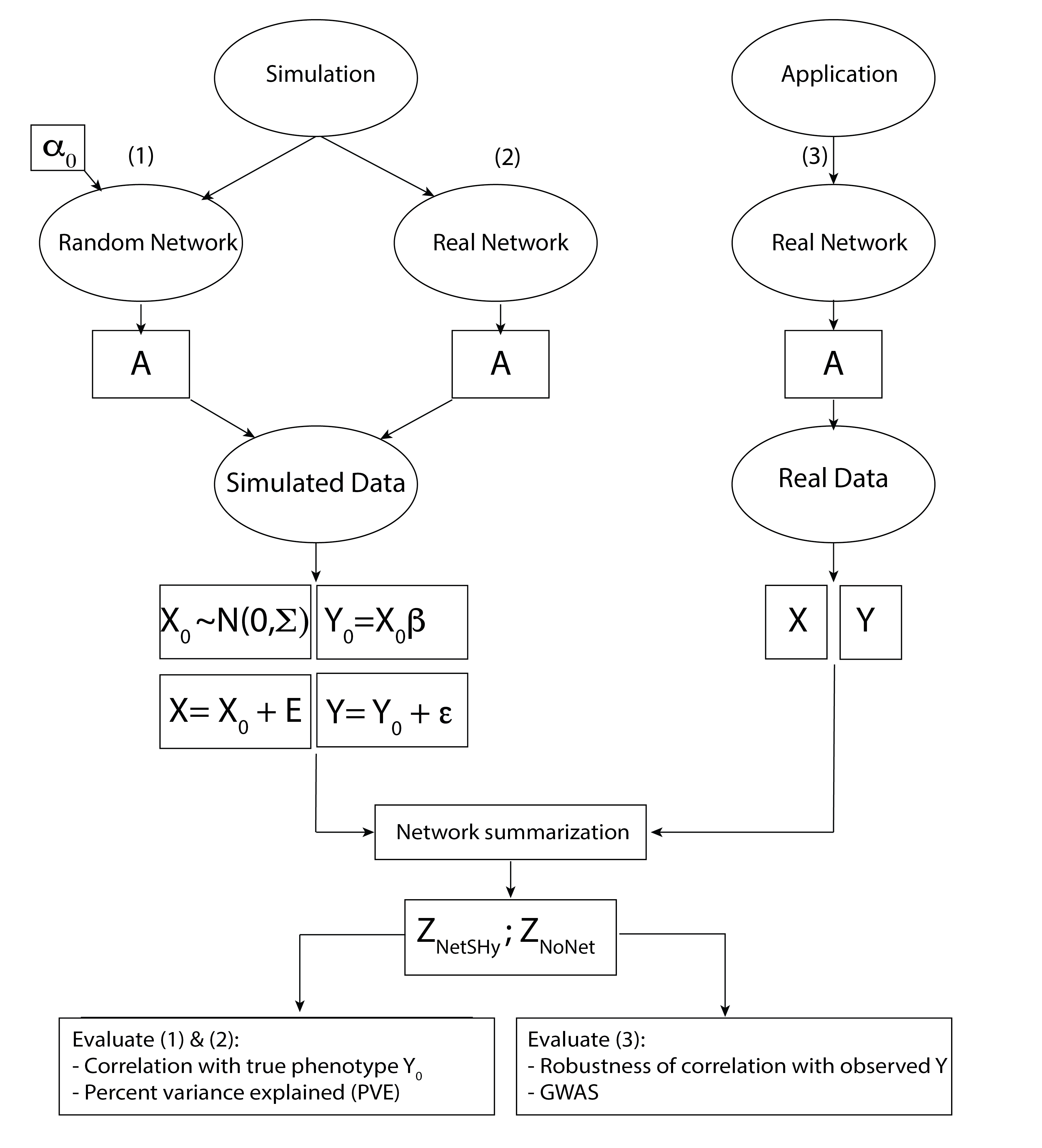

Supplement: btac818_Supplementary_Data [file btac818_supplementary_data.zip › Fig_S2.png]

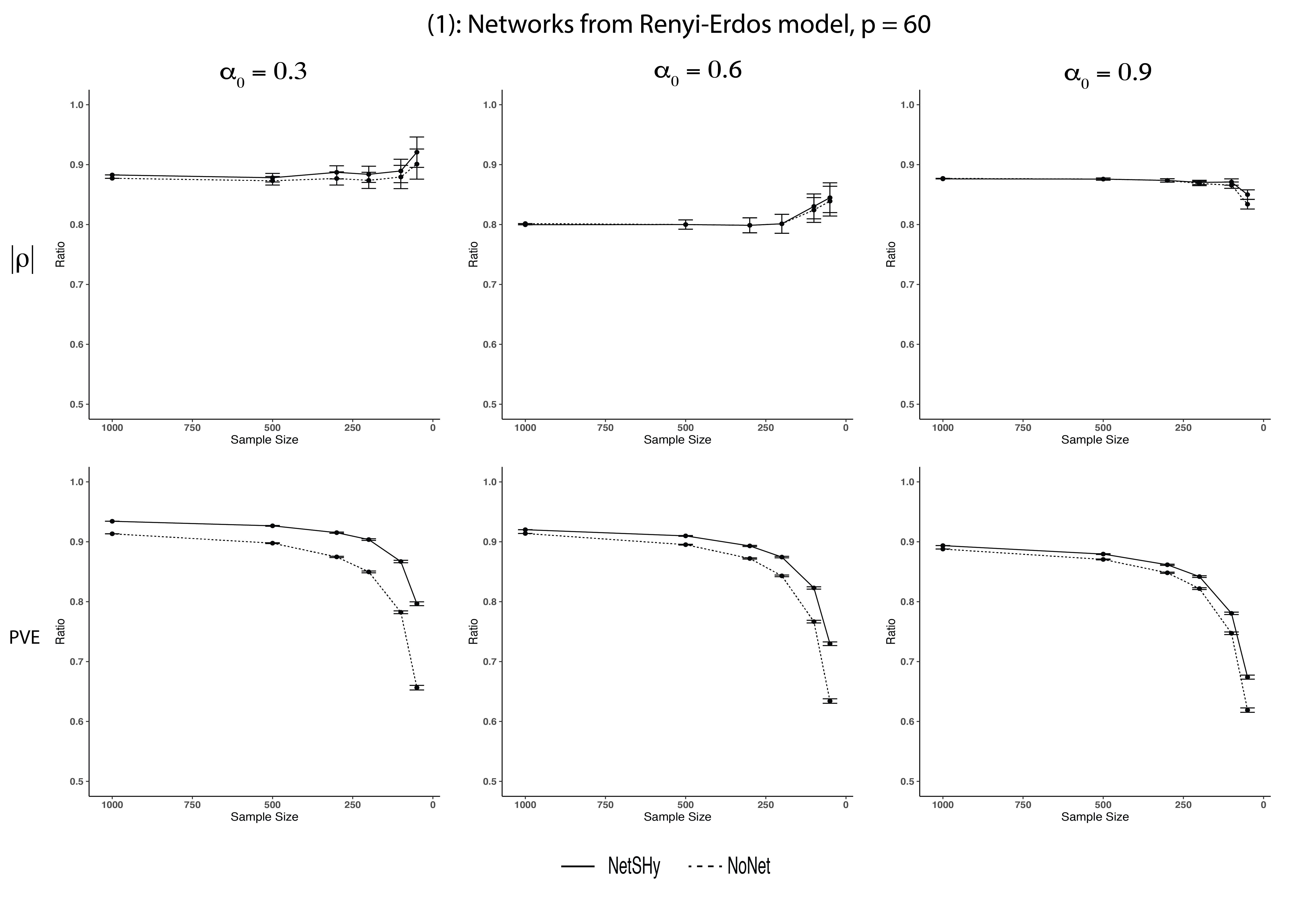

Supplement: btac818_Supplementary_Data [file btac818_supplementary_data.zip › Fig_S3.png]

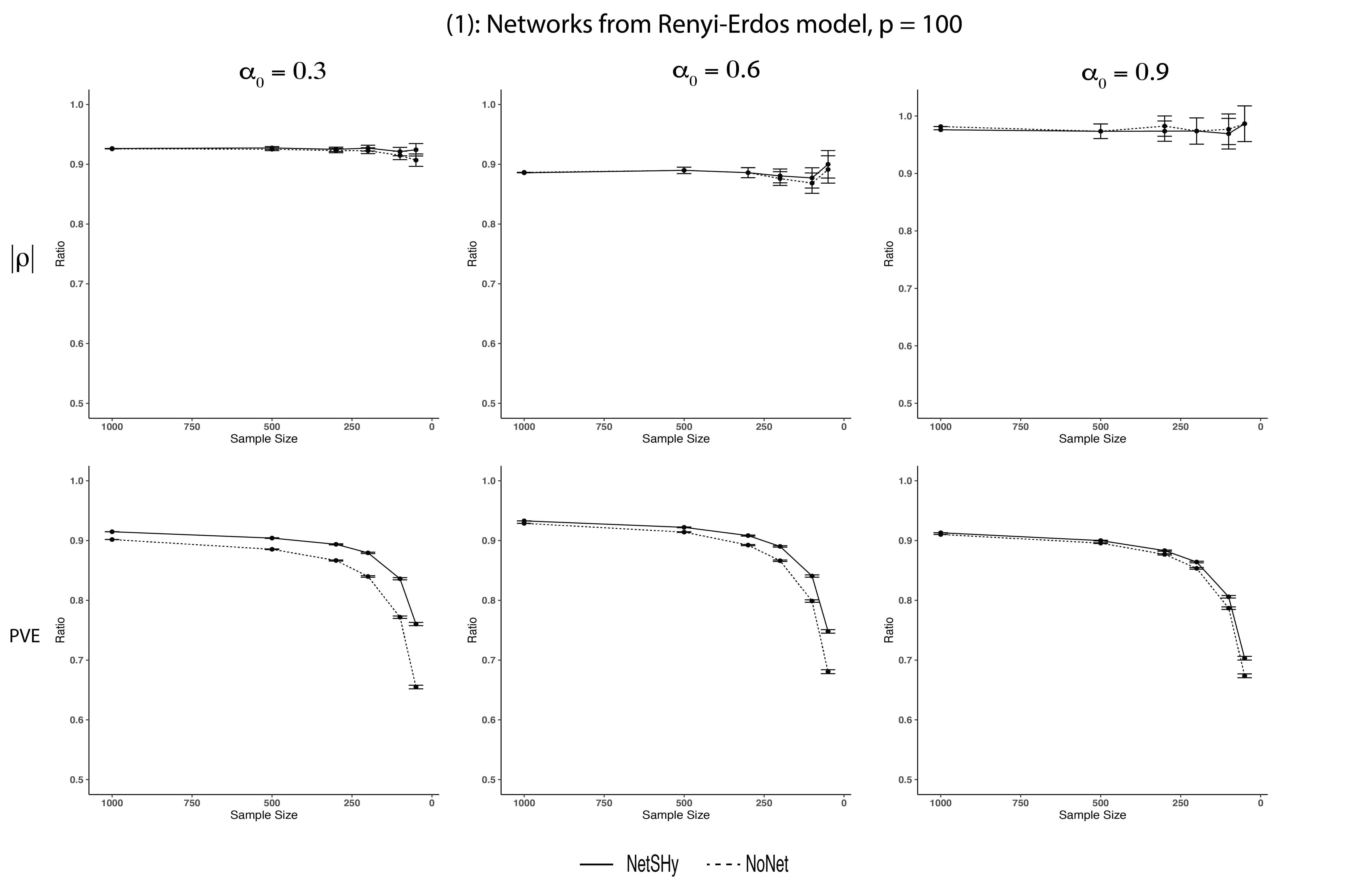

Supplement: btac818_Supplementary_Data [file btac818_supplementary_data.zip › Fig_S4.png]

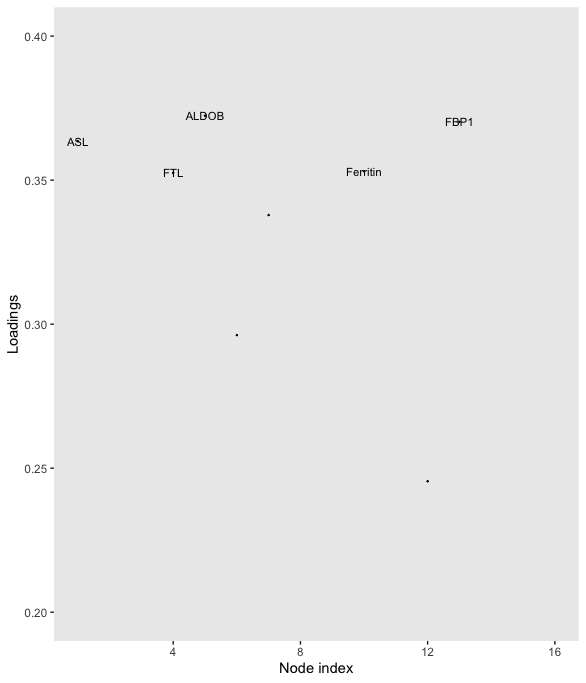

Supplement: btac818_Supplementary_Data [file btac818_supplementary_data.zip › Fig_S5.png]

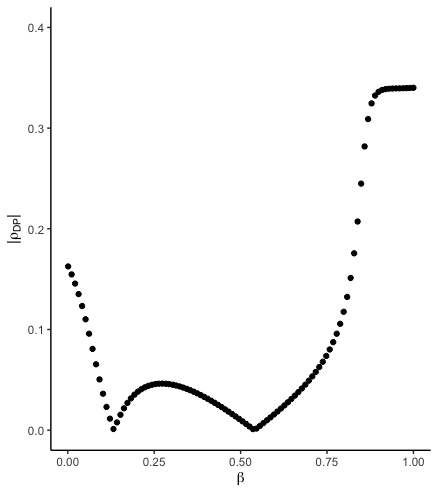

Supplement: btac818_Supplementary_Data [file btac818_supplementary_data.zip › Fig_S6.png]

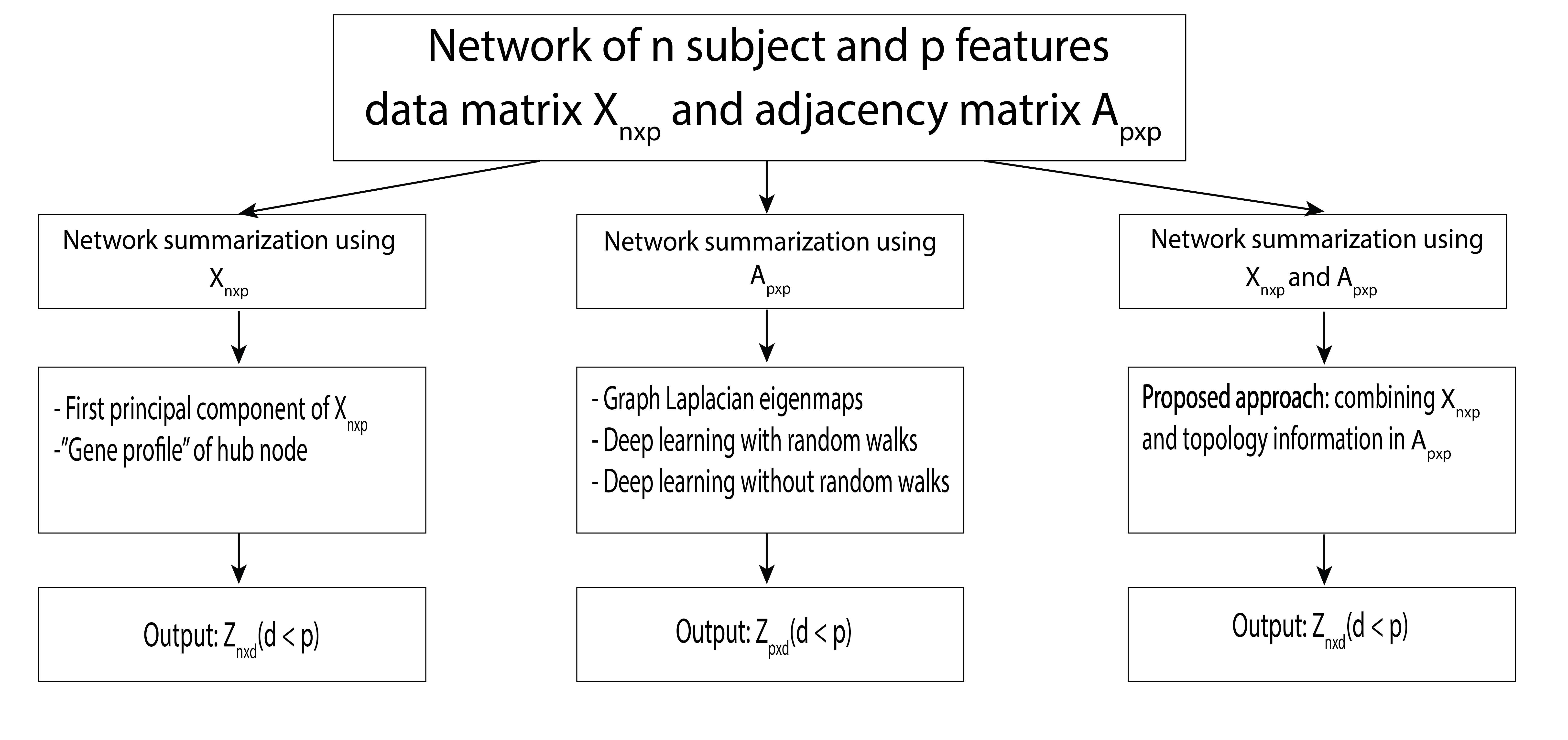

Supplement: btac818_Supplementary_Data [file btac818_supplementary_data.zip › Fig_S1.png]
